# Successful management of recalcitrant fungal keratitis with topical caspofungin

**DOI:** 10.3205/oc000206

**Published:** 2022-07-18

**Authors:** Siddhi Goel, Arpit Sharma, Rajesh Sinha

**Affiliations:** 1 Dr. Rajendra Prasad Centre for Ophthalmic Sciences, All India Institute of Medical Sciences, New Delhi, India

**Keywords:** caspofungin, recalcitrant fungal keratitis, echinocandins, fungal corneal ulcer, corneal ulcer healing

## Abstract

**Objective:**

To report a case of recalcitrant fungal keratitis successfully managed with topical caspofungin acetate in North India.

**Methods:**

Case report and literature review.

**Results:**

An 18-year-old male patient presented with complaints of redness, watering, pain and diminution of vision in the right eye and was referred to our centre as a case of corneal ulcer. The patient was examined and found to have a near total epithelial defect, with corneal infiltrates approximately 8x8 mm. A provisional diagnosis of polymicrobial keratitis was made based on corneal scraping suggestive of *Staphylococcus aureus* and confocal scan revealing fungal hyphae. The patient failed to respond to topical voriconazole 1%, natamycin 5% and moxifloxacin hydrochloride 0.5% with oral voriconazole. In view of the poor response to these drugs, the patient was subsequently administered topical caspofungin 0.5% in place of natamycin and continued on topical and oral voriconazole, in addition to topical moxifloxacin and cycloplegics, which finally led to healing with minimal scarring. The patient attained a best corrected visual acuity (BCVA) of 6/12.

**Conclusion:**

Topical caspofungin may be used as a useful and effective alternative in cases of recalcitrant fungal keratitis. It may result in healing with minimal scarring.

## Introduction

Fungal keratitis is a significant cause of ocular morbidity, especially in the developing countries, resulting in sequelae leading to irreversible corneal blindness. However, the treatment of fungal keratitis remains challenging, with natamycin being the only commercially available antifungal eyedrop. Herein, we report a case of recalcitrant fungal keratitis which was successfully managed with topical caspofungin acetate 0.5%.

## Case description

**Figure 1:**
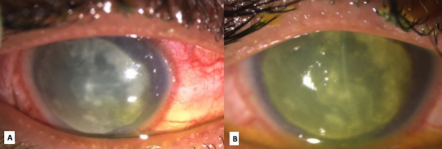
Clinical photograph on diffuse slit lamp illumination of the right eye at presentation showing (A) a large 8x8 mm infiltrate with circumcorneal congestion and lid edema; (B) after staining with 2% sodium fluorescein, note the near total epithelial defect.

**Figure 2:**
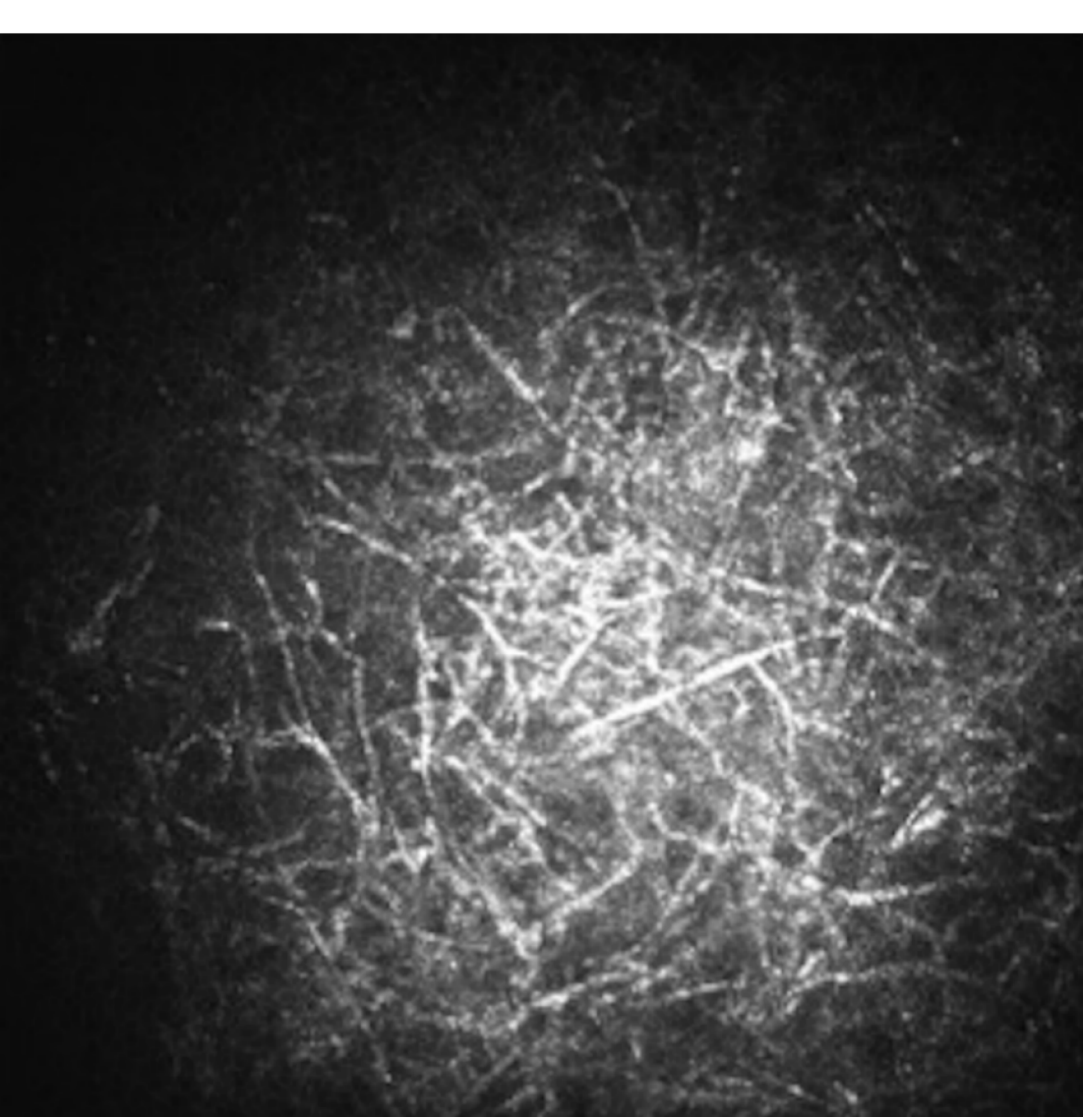
Confocal image of the patient showing branching hyphae suggestive of fungal keratitis.

An 18-year-old male patient with a history of redness, pain, watering, foreign body sensation and diminution of vision in his right eye for the past month was referred to us. The patient had a prior history of trauma to the right eye with dust particles, subsequent to which he had put honey in the eye for symptomatic relief. The application of honey in the eye could be a source of keratitis. On presentation, the Snellen visual acuity was hand motion close to the face with accurate projection of rays in the right eye and 6/6 in the left eye. Slit-lamp examination revealed a near total epithelial defect with corneal infiltrates of 8x8 mm extending up to ½ of corneal thickness with circumciliary congestion and lid edema (Figure 1 (Fig. 1)). Corneal scrapings were taken for smear and culture, and *Staphylococcus aureus* was isolated in bacterial culture, which was sensitive to moxifloxacin, gatifloxacin, cefazolin, and vancomycin. Confocal microscopy was performed, which revealed the presence of fungal hyphae (Figure 2 (Fig. 2)). Posterior segment examination on ultrasonography did not reveal any evidence of presence of vitreous exudates or retinal detachment. Treatment was started with topical voriconazole 1% (prepared my mixing 20 mL ringer lactate to 200 mg voriconazole lyophilised powder), natamycin 5% (commercially prepared eye drops), vancomycin 5% (500 mg of vancomycin powder was reconstituted with 2 mL sterile water and then added to 8 mL of artificial tears), administered every hour with homatropine hydrobromide 2% QID and oral voriconazole at a dosage of 200 mg twice daily. After 3 days, the lesion had not progressed, but there was no change in corneal infiltration or epithelial defect, and the patient had worsened symptomatically. The culture report was negative for fungus and *Acanthamoeba*. At one week, there was a further increase in symptoms and no reduction in infiltrates. The patient was subsequently switched from concentrated vancomycin to topical 0.5% moxifloxacin. The epithelial defect remained unchanged. Meanwhile, a corneal scrape GeneXpert sample to rule out atypical mycobacterium also turned out to be negative. GeneXpert is a cartridge-based nucleic acid amplification test (NAAT) for rapid diagnosis of tuberculosis and atypical mycobacteria. In view of the poor response with clinical worsening on day 14 of starting the treatment, we decided to replace topical natamycin with topical caspofungin acetate 0.5%, applied every hour.

**Figure 3:**
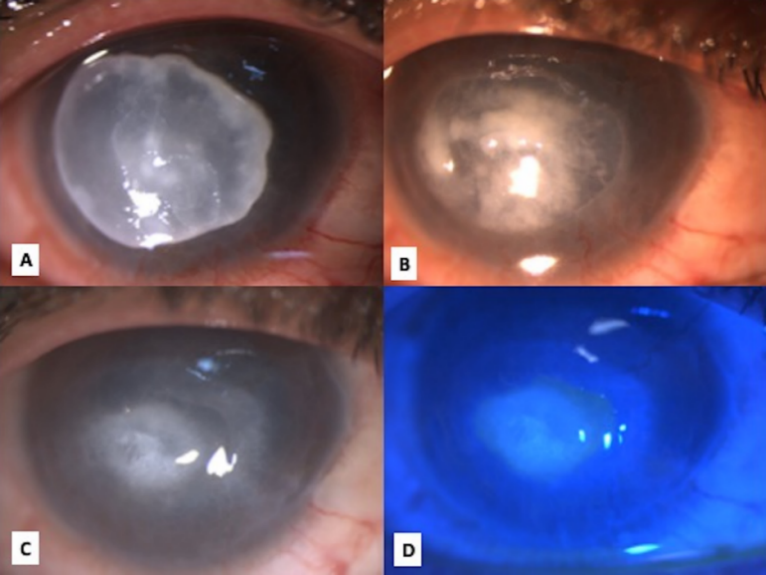
Clinical photograph on diffuse slit lamp illumination of the right eye (A) on day 7 of starting topical caspofungin; note the reduction in size of infiltrate with evidence of vascularisation suggesting healing; (B) on day 14, further reduction in ulcer size; (C) on day 28, note the evidence of scarring; (D) on cobalt blue illumination showing a residual epithelial defect of 3x2 mm.

**Figure 4:**
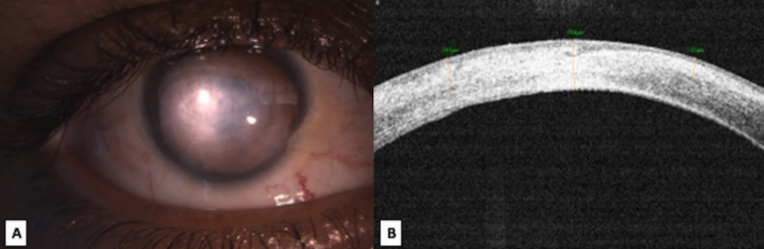
Clinical photograph on diffuse slit lamp illumination of the right eye (A) at 6 weeks, showing complete healing of the ulcer with residual stromal scarring; (B) anterior segment optical coherence tomography (ASOCT) showing residual stromal scarring after complete healing.

To prepare the eyedrops, 1 vial of 50 mg of caspofungin acetate was diluted in 10.5 mL of sterile normal saline; all eyedrops were freshly prepared weekly, kept at 4°C, and protected from light. Three days later, clinical improvement was observed. Following the commencement of the treatment, healing of the corneal epithelium and resolution of the corneal infiltrate were observed from the periphery towards the centre with evidence of vascularisation, and the patient showed commendable clinical response (Figure 3 (Fig. 3)). At 6 weeks of follow-up, the ulcer had healed completely. Although the patient initially had limbus to limbus corneal infiltrates, the scarring was less dense and involved superficial cornea with relative peripheral corneal sparing (Figure 4 (Fig. 4)). The patient attained an uncorrected visual acuity of 6/36 in the right eye, which improved to 6/12 with a trial of rigid gas permeable (RGP) contact lens.

## Discussion

Fungal keratitis accounts for nearly 50% of cases of keratitis in the developing countries and tropical regions [1], [2]. *Aspergillus* is the most frequent cause of fungal keratitis in our region, followed by *Fusarium* [3]. Vegetative particle injury as well as use of homemade medicines and immunosuppression have been found to be the major predisposing risk factors for fungal keratitis. Both these organisms are known to respond well to a combination treatment of natamycin and voriconazole.

Currently marketed echinocandins comprise caspofungin, micafungin and anidulafungin [4]. They are high molecular weight lipopeptides. Caspofungin is an echinocandin with excellent in vitro activity against several fungi like *Aspergillus*, and even *Candida* sp. [5]. It inhibits the synthesis of (1,3)-D-glucan, an essential component of the fungal cell wall leading to cell lysis. This enzyme is unique to fungi, thus, systemic toxicity of caspofungin is significantly less than that with the azoles.

To the best of our knowledge, there have only been two reports of the use of topical caspofungin in fungal keratitis in humans [6], [7]. The use of topical caspofungin has been studied in rabbits [8]. Tu et al. [6] reported a case of refractory alternaria keratitis in the US, which did not respond to topical natamycin and was successfully managed with topical, oral and intrastromal voriconazole along with topical caspofungin 0.5%. The patient attained a best spectacle corrected visual acuity (BSCVA) of 20/25. Hurtado-Sarrió et al. [7] reported a case of a 60-year-old female patient from Spain who developed a post-penetrating keratoplasty infection with *Candida albicans*. She failed to respond to topical and oral voriconazole, consequent to which she was started on topical caspofungin 1 hourly. Clinical improvement and negative cultures were noted after 1 week of starting caspofungin. In the present case, there was a marked response which started as early as 3 days after initiation of treatment in terms of symptomatic relief and objective reduction in the density and area of infiltrates. At the end, the ulcer which had involvement of the whole cornea resolved completely with a good corneal clarity in the peripheral part and little haze in the central part of the cornea.

## Conclusion

In conclusion, topical caspofungin 0.5% is a new and promising option for the management of refractory fungal keratitis with no evidence of ocular toxicity. In our case, we observed a unique pattern of healing from the periphery to the centre with minimal scarring, However, future studies with larger samples are required to further evaluate its efficacy and tolerance.

## Notes

### Competing interests

The authors declare that they have no competing interests.
